# Reflex Stimulation of the Vagus Centre

**Published:** 1912-12

**Authors:** 


Reflex Stimulation of the Vagus Centre, A. T. Brand, M. D., C. M., *Caledonian Med. Jour.*, Oct., 1912. Linimentum iodi is painted over the course of the vagus in the neck. Good results are reported for exophthalmic goitre, spasmodic asthma, pertussis and nausea and vomiting generally.

